# Dysphagia in frail elderly: self-reported mealtime symptoms and risk

**DOI:** 10.11606/s1518-8787.2025059006440

**Published:** 2025-08-25

**Authors:** Cirley Novais Valente, Fernanda Chiarion Sassi, Ana Paula Ritto, Isadora Cardoso Salles Fila Pecenin, Claudia Regina Furquim de Andrade

**Affiliations:** I Universidade de São Paulo. Faculdade de Medicina. Departamento de Fisioterapia, Fonoaudiologia e Terapia Ocupacional. São Paulo, SP, Brasil; II Universidade de São Paulo. Escola de Enfermagem. São Paulo, SP, Brasil

**Keywords:** Frail Elderly, Frailty, Deglutition Disorders, Speech-Language Pathology

## Abstract

**OBJECTIVE:**

This study aimed to characterize swallowing and investigate the association between self-reported coughing and/or choking during meals and the risk of dysphagia in frail elderly individuals receiving healthcare at a specialized center in São Paulo, Brazil.

**METHODS:**

This cross-sectional observational study included elderly individuals attending an Elderly Health Reference Unit (URSI) from July 2017 to December 2023. Data from all patients referred to the URSI were collected, excluding only those with incomplete medical records. The study proceeded in two phases: the first phase involved physical, functional, and social assessments, while the second phase included speech and swallowing evaluations. Participants were categorized into groups based on self-reported mealtime symptoms. Descriptive and inferential analyses compared these groups using the Mann-Whitney U test for quantitative data and Pearson's χ^2^ test for qualitative data. Risk analysis employed multiple logistic regression with forward stepwise selection.

**RESULTS:**

The study comprised 1,027 elderly individuals (mean age 78 years; 68.2% women; 64.2% self-identified as white). Approximately half reported frequent choking during meals. Sex, Multidimensional Assessment of the Elderly in Primary Care classification and score, polypharmacy, chronic diseases, falls, urinary incontinence, osteoporosis, cardiac disease, last dental visit, speech difficulty, change in food consistency, hearing loss, and hearing aid use were associated with mealtime symptoms. Speech alterations increased bronchoaspiration risk by 16%, memory complaints by 11%, and xerostomia and food consistency changes by 8%.

**CONCLUSION:**

Speech alterations, changes in food consistency, memory complaints, and xerostomia were identified as factors increasing the risk of bronchoaspiration. Early identification and a multidisciplinary approach to swallowing disorders in frail elderly individuals are crucial for preventing aspiration pneumonia and maintaining quality of life. These findings underscore the significance of proactive management strategies in clinical practice.

## INTRODUCTION

As demographic projections indicate a global aging trend driven by increased longevity and declining fertility rates^1^, Brazil is expected to see approximately one-third of its population aged 60 years or older by 2060^2^. This demographic shift requires the development and sustainability of health programs and services that enhance the physical and psychosocial well-being of elderly individuals.

Aligned with the National Health Policy for the Elderly in Brazil, established by Ordinance No. 2,528/2006^3^, the Municipal Health Department of São Paulo has implemented the Elderly Health Care Network (Rede de Atenção à Saúde da Pessoa Idosa — RASPI). RASPI aims to provide comprehensive care for the elderly through services encompassing promotion, prevention, diagnosis, treatment, rehabilitation, and maintenance of health, with a focus on integration with families and communities. A key strategy of RASPI is the Multidimensional Assessment of the Elderly in Primary Care (Avaliação Multidimensional da Pessoa Idosa na Atenção Básica — AMPI-AB), which classifies elderly individuals as healthy, pre-frail, or frail, directing their care either to Basic Health Units (Unidades Básicas de Saúde — UBS) at the primary level or specialized services such as Health Reference Units for the Elderly (Unidades de Referência à Saúde do Idoso — URSI) based on their specific needs^4^.

Frailty among the elderly is characterized by a loss of physical function or the accumulation of multiple deficits, influenced by biological, psychological, and social factors^5^. Its prevalence varies widely, from 4 to 59%, with higher rates observed in older individuals (> 85 years), females, those with chronic diseases, poor nutritional status, and disadvantaged socioeconomic backgrounds^5^. In Brazil, frailty prevalence among the elderly community stands at approximately 9.1%^6^. Variability in prevalence is attributed to differing definitions, diagnostic criteria, cohort demographics, and environmental and social factors^5^. Frail older adults are predisposed to falls, difficulties in performing Instrumental Activities of Daily Living (IADLs), cognitive decline^6^, and swallowing disorders^7^.

The aging process significantly impacts swallowing, and this becomes evident in presbyphagia, which refers to changes in the swallowing mechanism observed in healthy elderly individuals due to normal aging. These changes, including impaired bolus preparation and increased pharyngeal residue, can progress to oropharyngeal dysphagia when compounded by factors such as reduced muscle mass^8^. Dysphagia adversely affects morbidity, mortality, and hospitalization duration among the elderly, with high prevalence rates reported in hospital settings (36%), rehabilitation services (42%), and Long-Term Care Facilities (LTCFs) (50%)^9^, particularly among older individuals and females^7^. Conditions like dementia, stroke, and Parkinson's disease exacerbate dysphagia, with prevalence rates exceeding 85% in advanced dementia and varying from 8.1 to 80% post-stroke^10^.

Early identification of dysphagia in frail elderly individuals is crucial to prevent complications such as aspiration pneumonia, reduce hospitalization durations, minimize readmission rates, and lower mortality^7^. However, clinical assessment and rehabilitation for complex cases require specialized instruments and skills, posing challenges in primary care settings. Validated self-perception health questions serve as practical, comprehensive, speedy, and cost-effective screening tools in various contexts^11^, facilitating early detection of dysphagia risk by multidisciplinary professionals, appropriate referrals, and tailored interventions. Implementing this approach can significantly improve quality of life for frail elderly individuals. Therefore, this study aimed to investigate the correlation between self-reported coughing or choking during meals and the risk of dysphagia in frail elderly individuals treated at a specialized center in São Paulo, Brazil.

## METHODS

### Study Design

This cross-sectional observational study focuses on elderly individuals from the Lapa and Pinheiros regions in São Paulo, Brazil, using the URSI. Ethical approval was obtained from the Ethics Committee for Research Project Analysis at the Hospital das Clínicas of the Universidade de São Paulo School of Medicine (CAPPesq HCFMUSP, approval number 5,964,585). Data gathering only began after obtaining written Informed Consent Forms (ICFs). In cases where ICFs couldn't be obtained (e.g., due to lack of contact or deceased status), a Data Utilization Commitment Agreement was implemented.

### Study Location

The URSIs play a crucial role in São Paulo City's health initiatives under the Municipal Policy for Elderly Health Care, focusing on specialized treatment for complex diseases and specific health issues among the elderly. These facilities also prioritize preventive healthcare and health promotion. In São Paulo, there are 13 URSIs strategically located across six Regional Health Coordinators, with two units in the western region serving the communities of Butantã, Lapa, and Pinheiros^4^. Access to URSI services typically begins through the UBS, following the AMPI-AB. This assessment evaluates 17 parameters, including age, self-perceived health, chronic conditions, physical function, cognition, and activities of daily living, categorizing individuals as healthy, pre-frail, or frail. Referral criteria to URSI also include neuropsychiatric disorders, multiple chronic diseases, gait instability, and recurrent falls^12^.

### Study Sample

For this study, elderly individuals aged 60 years and older of both sexes were selected, referred to the Elderly Health Reference Unit Geraldo de Paula Souza (URSI GPS). The URSI GPS serves as a reference for 15 UBS under the Technical Health Supervision of Lapa/Pinheiros, within the West Regional Health Coordinator. Data were collected from clinical and speech therapy records in medical charts between July 2017 and December 2023, considering the initial assessment at the institution and the clinical evaluation of swallowing and risk of bronchoaspiration. Participants with incomplete data were excluded.

### Procedure

The investigation comprised two distinct phases. In phase 1, the functional attributes of the participants were characterized, alongside the identification of any swallowing-related complaints. Following this initial phase, appointments for evaluations in the second phase of the study were scheduled, with an average interval of two weeks between the two phases. Subsequently, in the second phase, the sample was categorized into two cohorts: participants reporting no swallowing complaints, and participants reporting swallowing complaints. Specific swallowing data were acquired by randomly selecting 50 individuals from each group (non-probabilistic sampling), who then underwent both the Specific Gerontological Assessment of Speech, Communication and Oral Functions and a clinical assessment of swallowing.

#### Phase 1: Characterization of Physical, Social, and Psychological Capacities

In the initial phase of this study, we assessed physical, social, and psychological capacities using the AMPI-AB score obtained at UBS as a referral criterion to the URSI. The Gerontological Global Assessment, a standardized instrument by the Municipal Health Secretariat's Elderly Health Technical Area for URSI use, facilitated initial data collection. The AMPI-AB score categorizes elderly individuals into low, intermediate, or high complexity care levels, defining functional categories: 0–5 points (healthy), 6–10 points (pre-frail), and ≥ 11 points (frail)^12^.

Variables extracted for sample characterization include gender, race/ethnicity, education, age, body mass index (BMI), polypharmacy, chronic diseases, smoking, chronic pain, falls, living arrangements, social support, motivation for activities of daily living (ADL), memory and hearing complaints, speech difficulties, dental visits, oral health issues, food consistency changes, and choking/coughing during meals. The latter serves as the pivotal question for stratifying participants into groups based on their response (yes/no)^13^.

#### Phase 2: Assessment of Speech and Swallowing Aspects

In the second phase of the study, we conducted an evaluation of participants’ speech and swallowing aspects, as follows:

- Specific Gerontological Assessment of Speech, Communication and Oral functions. This protocol comprises a set of tools designed for each professional category within the interdisciplinary team at the URSI. The Specific Gerontological Assessment (*Avaliação Gerontológica Específica—*AGE of Speech, Communication and Oral Functions was used to identify complaints or difficulties related to oral communication, feeding, dizziness, and hearing. The variables considered included comprehension of simple and complex commands, speech and language alterations, dysphonia, complaints of memory and attention deficits, feeding route, salivation, chewing, use of dentures, history of recent pneumonia, and presence of coughing, choking, and throat clearing before, during, or after meals, as well as complaints of dizziness and use of hearing aid devices (HAD)^13^;
- Preliminary Assessment Protocol (PAP). This protocol was used to describe and evaluate general, respiratory, speech, voice, orofacial and cervical structure aspects. Clinical measures considered in the analysis included: speech intelligibility, prosody, diadochokinesis, pneumophonoarticulatory coordination, gag reflex, cough quality, laryngeal elevation, saliva swallowing, dentition, and strength and mobility of lips, tongue, and cheeks^14^;
- Protocol for the Introduction and Transition of Feeding (PITF). This is a widely used tool by speech therapists in Brazil for the functional evaluation of swallowing. The protocol is based on the provision of foods and liquids of different consistencies and larger volumes, following the PAP. Its methodology incorporates the observation of signs and clinical symptoms suggestive of laryngotracheal penetration/aspiration and other signs commonly observed in the clinical practice of speech therapists working with dysphagia^15^.

To assess the functional level of swallowing, we utilized the Functional Oral Intake Scale from the American Speech-Language-Hearing Association National Outcome Measurement System (ASHA NOMS 2003). This multidimensional scale assigns a number from 1 to 7 based on the supervision needed for feeding and the diet level^16^:

- Level 1: Unable to safely swallow orally; receives all nutrition and hydration through alternative feeding routes;
- Level 2: Unable to safely swallow orally for nutrition and hydration; may ingest some consistency in therapy with maximum cues; alternative feeding route required;
- Level 3: Ingests less than 50% of nutrition orally; safe swallowing with moderate cue use or maximum diet restrictions; alternative feeding route necessary;
- Level 4: Safe swallowing with moderate cue use; moderate diet restrictions; alternative feeding route or oral supplement still required;
- Level 5: Safe swallowing with minimal diet restrictions; minimal cue use occasionally; self-monitors during meals;
- Level 6: Independent eating and drinking; minimal cue use; self-monitors when needed; specific food items may need avoidance; extended feeding time possible;
- Level 7: Independent feeding; safe and efficient swallowing across all consistencies; effective use of compensatory strategies.

**Figure 1:**
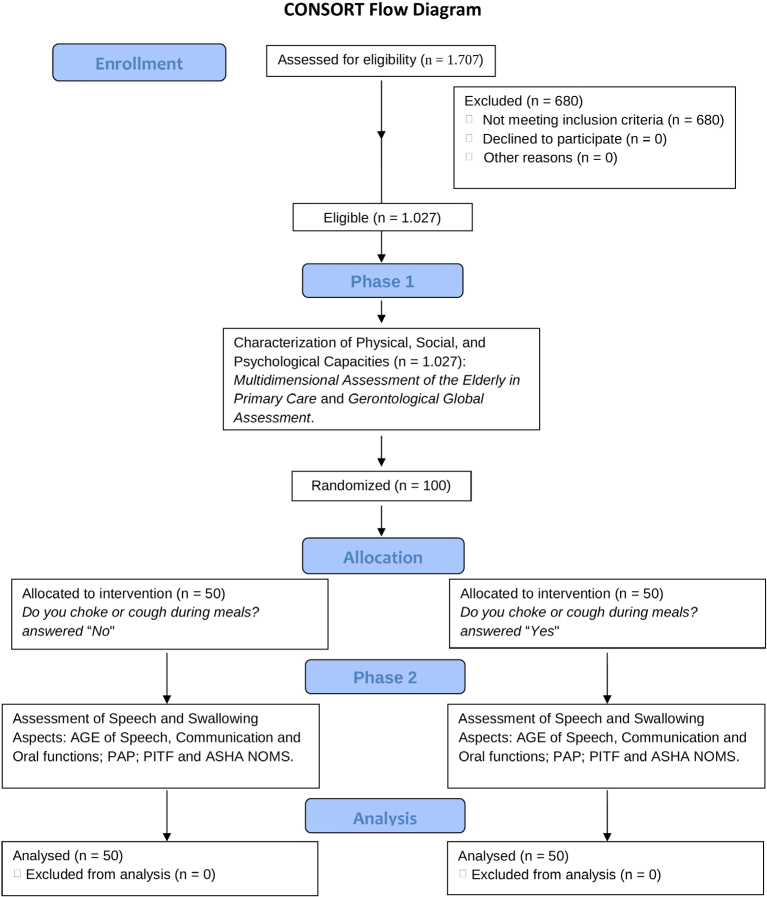
CONSORT flow diagram.

The ASHA NOMS scale categorizes individuals based on their ability to safely swallow and manage diets, aiding in treatment planning and monitoring of dysphagia interventions. The CONSORT flow diagram detailing the progress of participants throughout the study is presented in Figure 1.

### Data Analysis

The data analysis used Statistical Package for the Social Sciences (SPSS) software version 27. The sample comprised 1,027 patients divided into two groups, based on self-reported coughing or choking during meals: 592 patients (57.6%) without and 435 patients (42.4%) with this complaint. Descriptive and inferential analyses employed the Mann-Whitney U test for quantitative data and Pearson's χ^2^ test for qualitative data, justified by non-normal data distribution confirmed by the Kolmogorov-Smirnov test. A significance level of 5% was applied.

Additionally, a subgroup of one hundred randomly selected patients underwent similar analysis methods. They were categorized based on objective swallowing assessments at the AGE of Speech, Communication and Oral functions: 61 patients without bronchoaspiration signs and 39 with signs. Descriptive statistics compared these groups.

Risk analysis for bronchoaspiration identified significant variables (p ≤ 0.05) from bivariate analyses with clinical relevance. These were included in a multiple logistic regression model using forward stepwise selection (5% entry and exit criteria) to assess associations with the negative outcome.

## RESULTS

### Global Gerontological Assessment

Between July 2017 and December 2023, URSI-GPS enrolled a total of 1,707 elderly individuals. Exclusions totaled 680 due to incomplete medical records (≥ 25%). The study included 1,027 participants aged 60 to 103 years (mean 78 years; standard deviation—SD = 8.5), predominantly female (68.2%), white (64.2%), with primary education (27.4%). Using AMPI-AB, 30.7% were classified as frail, averaging four chronic diseases (range: 0–12; SD = 1.9) and 5.6 medications (range: 0–20; SD = 3.4). Mean BMI was 26 (range: 11.8–47.6; SD = 5.4), indicating overweight tendency.

Participants were stratified by self-reported cough/choking during meals into "no swallowing complaints" and "swallowing complaints" groups. Significant differences (p < 0.05) were found in sex, AMPI-AB classification/score, chronic diseases, polypharmacy, assistive device use, hearing/speech difficulties, falls, food consistency changes, osteoporosis, heart disease, and urinary incontinence (Table 1).

**Table 1:** Intergroup comparison of demographic variables and medical record data

Variable		No swallowing complaints (n = 592)	Swallowing complaints (n = 435)	Total (n = 1,027)	p-value
**Age, in years**					
	n	592	435	1,027	0.281
	mean (SD)	77.6 (8.5)	78.7 (8.5)	78 (8.5)	
	median (min; max)	78 (60; 102)	79 (60; 103)	79 (60; 103)	
**Sex, n (%)**					
	Male	205 (34.6%)	122 (28%)	327 (31.8%)	0.046^a^
	Female	387 (65.4%)	313 (72%)	700 (68.2%)	
**Self-reported race/ethnicity, n (%)**					
	Black	25 (4.2)	31 (7.1)	56 (5.4)	0.359
	White	382 (64.5)	278 (64)	660 (64.2)	
	Mixed	76 (12.9)	48 (11)	124 (12.1)	
	Asian	27 (4.6)	16 (3.7)	43 (4.2)	
	Indigenous	2 (0.3)	1 (0.2)	3 (0.3)	
	Undeclared	80 (13.5)	61 (14)	141 (13.8)	
**Educational level, n (%)**					
	No formal education / illiterate	25 (4.2)	10 (2.3)	35 (3.4)	0.613
	Primary education	156 (26.4)	125 (28.7)	281 (27.4)	
	Elementary education	94 (15.9)	67 (15.4)	161 (15.7)	
	Secondary education	80 (13.5)	55 (12.7)	135 (13.1)	
	Higher/university education	138 (23.3)	101 (23.2)	239 (23.3)	
	Undeclared	99 (16.7)	77 (17.7)	176 (17.1)	
**AMPI-AB classification, n (%)**					
	Healthy	29 (4.9)	8 (1.9)	37 (3.6)	< 0.001^a^
	Pre-fragile	187 (31.6)	108 (24.8)	295 (28.7)	
	Fragile	151 (25.5)	164 (37.7)	315 (30.7)	
	Unavailable	225 (38)	155 (35.6)	(380 37)	
**AMPI-AB score**					
	n	367	280	647	<0.001^b^
	mean (SD)	6 (5.2)	7.1 (5.7)	6.8 (5.4)	
	median (min; max)	7 (0; 17)	9 (0; 17)	8 ((0; 17)	
**Number of chronic diseases**					
	n	592	435	1027	< 0.001^b^
	mean (SD)	4 (2)	4 (1.9)	4 (1.9)	
	median (min; max)	4 (0; 12)	4 (0; 10)	4 (0; 12)	
**Polypharmacy**					
	n	592	435	1,027	< 0.001^b^
	mean (SD)	5.2 (3.4)	6.1 (3.6)	5.6 (3.4)	
	median (min; max)	5 (0; 17)	6 (0; 20)	6 (0; 20)	
**BMI**					
	n	343	256	598	0.486
	mean (SD)	25.8 (5.3)	26.2 (5.5)	26 (5.4)	
	median (min; max)	25.2 (11.8; 47.6)	25.7 (14.6; 46.3)	25.4 (11.8; 47.6)	
**Living alone**					
	n (%)	137 (23.1)	98 (22.5%)	235 (22.9%)	0.733
**Absence of social support**					
	n (%)	156 (26.3)	116 (26.6)	272 (26.5)	0.664
**Smoking, n (%)**					
	No, never	294 (49.7)	229 (52.6)	593 (50.9)	0.551
	No, quit	231 (39)	168 (38.6)	399 (38.9)	
	Yes	63 (10.6)	36 (8.3)	99 (9.6)	
	Unanswered	4 (0.7)	2 (0.5)	6 (0.6)	
**Alcoholism, n (%)**					
	No, never	336 (56.7)	236 (54.3)	572 (55.7)	0.342
	No, quit	104 (17.6)	95 (21.8)	199 (19.4)	
	Yes	142 (24)	95 (21.8)	237 (23.1)	
	Unanswered	10 (1.7)	9 (2.1)	19 (1.8)	
**Memory complaint**					
	n (%)	247 (41.7)	195 (44.8)	442 (43)	0.515
**Chronic pain**					
	n (%)	405 (68.4)	310 (71.2)	715 (69.6)	0.430
**Disabling pain**					
	n (%)	191 (32.2)	165 (38)	356 (34.7)	0.076
**Use of assistive devices**					
	n (%)	275 (46.4)	238 (54.7)	513 (50)	0.031^a^
**Complaints of hearing loss**					
	n (%)	229 (38.6)	207 (47.5)	436 (42.5)	0.004^a^
**Complaints of speech difficulty**					
	n (%)	120 (20.2)	143 (32.8)	263 (25.6)	< 0.001^a^
**Falls in the past 12 months**					
	n (%)	312 (52.7)	263 (60.4)	575 (56)	0.046^a^
**Time since last dental visit, n (%)**					
	< 1 year	204 (34.4)	164 (37.37)	368 (35.8)	0.577
	1 to 2 years	130 (22)	103 (23.7)	233 (22.7)	
	3 years or more	195 (33)	133 (30.6)	328 (32)	
	Never went to the dentist	4 (0.7)	1 (0.2)	5 (0.5)	
	Does not know	59 (9.9)	34 (7.8)	93 (9)	
**Change in food consistency**					
	n (%)	139 (23.4)	138 (31.7)	277 (27)	0.012^a^
**Presence of oral wounds**					
	n (%)	32 (5.4)	33 (7.5)	65 (6.3)	0.244
**Diabetes mellitus**					
	n (%)	189 (31.9)	159 (36.5)	348 (33.9)	0.068
**High blood pressure**					
	n (%)	340 (57.4)	275 (63.2)	615 (59.9)	0.054
**Osteoporosis**					
	n (%)	99 (16.7)	92 (21.1)	191 (18.6)	0.048^a^
**Parkinson's disease**					
	n (%)	23 (3.8)	27 (6.2)	50 (4.9)	0.059
**High cholesterol**					
	n (%)	209 (35.3)	177 (40.6)	386 (37.6)	0.063
**Cardiac disease**					
	n (%)	140 (23.6%	130 (29.8)	270 (26.3)	0.024^a^
**Ophthalmic disease**					
	n (%)	153 (25.8)	135 (31)	288 (28)	0.051
**Urinary incontinence**					
	n (%)	211 (35.6)	218 (50.1)	429 (41.8)	< 0.001^a^
**Depressive symptoms**					
	n (%)	163 (27.5)	140 (32.1)	303 (29.5)	0.272
**Cognitive impairment**					
	n (%)	120 (20.2)	107 (24.6)	227 (22.1)	0.109

n: number of participants; SD: standard deviation; min.: minimum; max.: maximum; AMPI-AB: Multidimensional Assessment of the Elderly in Primary Care; BMI: body mass index.

a Significant difference according to Pearson's χ^2^ test;

b significant difference according to Mann-Whitney's U test.

### Specific Gerontological Assessment: AGE of Speech, Communication and Oral Functions

In the second stage of the study, a detailed analysis was conducted on the gerontological assessment using the AGE of Speech, Communication and Oral Functions and the clinical assessment of swallowing using the PAP and the Protocol for the Introduction and Transition of Feeding (PITF) evaluations of one hundred randomly selected participants. Table 2 presents a comparative analysis based on the data collected by the AGE. The results showed statistically significant differences (p < 0.05) between the groups in the following variables: speech and language alterations, salivation, eating meals too slowly, drowsiness after eating, history of recent pneumonia, dizziness, and use of HAD. The average score on the ASHA NOMS scale was 6.8 in the "no swallowing complaint" group (minimum = 4, maximum = 7) and 6.6 in the "swallowing complaint" group (minimum = 4, maximum = 7).

**Table 2:** Intergroup comparison of the results of the Specific Gerontological Assessment—AGE of Speech, Communication and Oral functions (n = 100).

Variable		No swallowing complaints (n = 50)	swallowing complaints (n = 50)	Total (n = 100)	p-value
**Speech and language alterations**					
	n (%)	5 (10)	14 (28)	19 (19)	0.048^a^
**Speech and language alterations (type), n (%)**					
	Dysarthria	3 (60)	11 (78.6)	14 (73.7)	0.430
	Aphasia	2 (40)	2 (14.2)	4 (21)	
	Acquired Apraxia of Speech	0 (0)	1 (7.2)	1 (5.3)	
**Dysphonia**					
	n (%)	43 (86)	44 (88)	87 (87)	0.950
**Level of dysphonia, n (%)**					
	Mild dysphonia	37 (86)	33 (75)	70 (80.5)	0.210
	Moderate dysphonia	4 (9.3)	10 (22.7)	14 (16.1)	
	Severe dysphonia	2 (4.7)	1 (2.3)	3 (3.4)	
**Complaints of attention deficits**					
	n (%)	37 (74)	41 (82)	78 (78)	0.334
**Complaints of memory deficits**					
	n (%)	38 (76)	37 (74)	75 (75)	0.817
**Salivation, n (%)**					
	Regular swallow	31 (62)	18 (36)	49 (49)	0.018^a^
	Accumulates and the swallows	1 (2)	4 (8)	5 (5)	
	Frequent choking	3 (6)	10 (20)	13 (13)	
	Xerostomia	13 (26)	11 (22)	24 (24)	
	Sialorrhea	2 (4)	7 (14)	9 (9)	
**Chewing difficulties**					
	n (%)	24 (48)	25 (50)	49 (49)	0.841
**Use of dentures**					
	n (%)	31 (62)	37 (74)	68 (68)	0.198
**Type of denture, n (%)**					
	Upper partial denture	7 (22.6)	11 (29.7)	18 (26.5)	0.257
	Lower partial denture	2 (6.4)	0 (0)	2 (2.9)	
	Complete denture	22 (71)	26 (70.3)	48 (70.6)	
**Eating meals too slowly**					
	n (%)	17 (34)	28 (56)	45 (45)	0.027^a^
**Throat clearing before, during, or after meals**					
	n (%)	15 (30)	17 (34)	32 (32)	0.668
**Drowsiness after eating**					
	n (%)	0	4 (8)	4 (4)	0.041^a^
**History of pneumonia in the past year**					
	n (%)	10 (20)	19 (38)	29 (29)	0.047^a^
**Dizziness**					
	n (%)	21 (42)	19 (38)	40 (40)	0.663
**Imbalance**					
	n (%)	8 (16)	10 (20)	18 (18)	0.603
**Use of Hearing Aid Devices**					
	n (%)	5 (10)	18 (36)	23 (23)	0.002^a^

n: number of participants; SD: standard deviation; min.: minimum; max.: maximum.

a Significant difference according to Pearson's χ^2^ test.

### Preliminary Assessment Protocol

The outcomes of the Preliminary Assessment Protocol (PAP) were evaluated and compared between the study groups, with the principal findings summarized in Table 3. Statistically significant differences (p < 0.05) were observed between the groups in the following parameters: temporal and spatial orientation, communicative initiative, speech intelligibility, vocal loudness, laryngeal elevation, and reduced mobility of the lips, tongue, and cheeks, as well as cheek strength.

**Table 3:** Intergroup comparison of the Preliminary Assessment Protocol (PAP) (n = 100).

Variable		No Swallowing Complaints (n = 50)	Swallowing Complaints (n = 50)	Total (n = 100)	p-value
**Temporal and spatial orientation**					
	n (%)	47 (94)	37 (74)	84 (84)	0.006^a^
**Communicative initiative**					
	n (%)	48 (96)	42 (84)	90 (90)	0.046^a^
**Speech unintelligibility**					
	n (%)	6 (12)	17 (34)	23 (23)	0.009^a^
**Voice pitch, n (%)**					
	High-pitched	1 (2)	2 (4)	3 (3)	0.504
	Low-pitched	0 (0)	1 (2)	1 (1)	
**Voice loudness, n (%)**					
	Soft	4 (8)	13 (26)	17 (17)	0.029^a^
	Loud	0 (0)	2 (4)	2 (2)	
**Lips, n (%)**					
	Reduced mobility	1 (2)	8 (16)	9 (9)	0.002^a^
	Reduced strength	19 (38)	29 (58)	48 (48)	0.068
**Tongue, n (%)**					
	Reduced mobility	5 (10)	2 (4)	7 (7)	0.015^a^
	Reduced strength	30 (60)	35 (70)	65 (65)	0.225
**Cheeks, n (%)**					
	Reduced mobility	0 (0)	12 (24)	12 (12)	< 0.001^a^
	Reduced strength	18 (36)	32 (64)	50 (50)	0.012^a^
**Reduced laryngeal elevation, n (%)**		25 (50)	39 (78)	64 (64)	0.004^a^

n: number of participants.

a Significant difference according to Pearson's χ^2^ test.

### Additional Analysis: Subgroups Defined by the Specific Gerontological Assessment — AGE of Speech, Communication and Oral Functions

Table 4 presents a comparative analysis based on data collected from the AGE of Speech, Communication and Oral Functions following the objective assessment of swallowing in the groups. Statistically significant differences (p < 0.05) were observed between patients without signs of bronchoaspiration and those with signs of bronchoaspiration in the following variables: degree of dysphonia, complaints of memory deficits, salivation, chewing difficulties, use of dentures, eating meals too slowly, drowsiness after eating, and use of HAD. The average score on the ASHA NOMS scale was 6.7 for both groups, with patients without signs of bronchoaspiration ranging from a minimum score of 4 to a maximum of 7, and patients with signs of bronchoaspiration ranging from a minimum score of 5 to a maximum of 7.

**Table 4:** Intergroup comparison (according to the signs of bronchoaspiration) based on data from the Specific Gerontological Assessment—AGE of Speech, Communication and Oral functions (n = 100).

Variable		No signs of bronchoaspiration (n = 61)	Signs of bronchoaspiration (n = 39)	Total (n = 100)	p-value
**Speech and language alterations**					
	n (%)	8 (13.1)	11 (28.2)	19 (19)	0.133
**Speech and language alterations (type), n (%)**					
	Dysarthria	4 (50)	10 (90.9)	14 (73.7)	0.122
	Aphasia	3 (37.5)	1 (9.1)	4 (21)	
	Acquired Apraxia of Speech	1 (12.5)	0 (0)	1 (5.3)	
**Dysphonia**					
	n (%)	52 (85.2)	35 (89.7)	87 (87)	0.505
**Level of dysphonia, n (%)**					
	Mild dysphonia	46 (88.5)	24 (68.6)	70 (80.5)	0.034^a^
	Moderate dysphonia	4 (7.7)	10 (28.6)	14 (16.1)	
	Severe dysphonia	2 (3.8)	1 (2.9)	3 (3.4)	
**Complaints of attention deficits**					
	n (%)	44 (72.1)	34 (87.2)	78 (78)	0.076
**Complaints of memory deficits**					
	n (%)	40 (65.6)	35 (89.7)	75 (75)	0.006^a^
**Salivation, n (%)**					
	Regular swallow	36 (59)	13 (33.3)	49 (49)	0.013^a^
	Accumulates and then swallows	1 (1.6)	4 (10.3)	5 (5)	
	Frequent choking	5 (8.2)	8 (20.5)	13 (13)	
	Xerostomia	16 (26.2)	8 (20.5)	24 (24)	
	Sialorrhea	3 (4.9)	6 (15.4)	9 (9)	
**Chewing difficulties**					
	n (%)	25 (41)	24 (61.5)	49 (49)	0.045^a^
**Use of dentures**					
	n (%)	37 (60.7)	31 (79.5)	68 (68)	0.049^a^
**Type of denture, n (%)**					
	Upper partial denture	11 (29.7)	7 (22.6)	18 (26.5)	0.305
	Lower partial denture	2 (5.4)	0 (0)	2 (2.9)	
	Complete denture	24 (64.9)	24 (77.4)	48 (70.6)	
**Eating meals too slowly**					
	n (%)	20 (32.8)	25 (64.1)	45 (45)	0.002^a^
**Throat clearing before, during, or after meals**					
	n (%)	18 (29.5)	14 (35.9)	32 (32)	0.504
**Drowsiness after eating**					
	n (%)	0 (0)	4 (10.3)	4 (4)	0.011^a^
**History of pneumonia in the past year**					
	n (%)	15 (24.6)	14 (35.9)	29 (29)	0.224
**Dizziness**					
	n (%)	23 (37.7)	17 (43.6)	40 (40)	0.558
**Imbalance**					
	n (%)	9 (14.8)	9 (23.1)	18 (18)	0.291
**Use of Hearing Aid Devices**					
	n (%)	9 (14.8)	14 (35.9)	23 (23)	0.014^a^

n: number of participants.

a Significant difference according to Pearson's χ^2^ test.

### Risk Analysis

Table 5 illustrates the multiple logistic regression analysis used to evaluate the association of various characteristics with the risk of bronchoaspiration. Only variables with significant results are presented. The analysis revealed that altered speech, changes in food consistency, choking, memory complaints, and xerostomia were significant risk factors for bronchoaspiration. Specifically, participants with speech and language alterations, such as dysarthria and/or apraxia, exhibited a 16% increased risk of bronchoaspiration. Those reporting complaints of memory deficits had an 11% higher risk, while the presence of xerostomia and changes in food consistency elevated the risk by 8% compared to those without these characteristics.

**Table 5:** Multivariate logistic regression model for prediction of risk of bronchoaspiration (resulting model).

Variable	Odds ratio	CI (95%)		p-value
		Lower	Upper	
**Speech and language alterations**	0.163	0.030	0.886	0.036^a^
**Change in food consistency**	0.085	0.015	0.487	0.005^a^
**Choking**	0.059	0.011	0.329	0.001^a^
**Complaints of memory deficits**	0.119	0.019	0.757	0.024^a^
**Xerostomia**	0.087	0.008	0.953	0.046^a^

CI: confidence interval.

a Statistically significant difference, according to multivariate logistic regression—ackward stepwise selection method.

## DISCUSSION

This study investigated the prevalence of choking during meals, along with associations between dysphagia and demographic and health factors, and the risk of bronchoaspiration among elderly individuals receiving care at an URSI in São Paulo's Western Zone, Brazil. Results revealed a 42% prevalence of choking, notably higher than reported in studies from Brazil (8.1%)^17^, the USA (20.4%)^18^, and Japan (25.1–53.8%)^19^. This difference may be attributed to the population's composition, predominantly frail elderly with multiple comorbidities, which are known to increase the prevalence of swallowing disorders^7^.

The results of phase 1 provide insights into the demographic and clinical characteristics of elderly individuals receiving care at the URSI-GPS. The higher prevalence of women and individuals with low educational levels aligns with the typical profile seen in public health services in Brazil, reflecting social vulnerabilities that may affect healthy aging. This is consistent with a Brazilian study that found women and individuals with lower education tend to assess their health more negatively, underscoring the role of socioeconomic factors in shaping elderly health perceptions^20^. Additionally, the analysis of self-reported choking and coughing during meals revealed significant associations with frailty, polypharmacy, chronic diseases, use of assistive devices, and falls. These findings are consistent with a study on elderly Japanese individuals, which demonstrated that dysphagia is linked to various aspects of frailty, including oral, physical, cognitive, and psychological dimensions. This highlights the complexity of dysphagia and the importance of considering frailty as a critical factor in the assessment and management of elderly individuals with swallowing difficulties^21^.

Elderly individuals with xerostomia exhibited an 8% increased risk of bronchoaspiration, corroborating the literature that associates dry mouth with impaired swallowing in older adults^22^. While a meta-analysis indicates a global prevalence of xerostomia at 33.37%, our study observed a lower rate of 22%^23^. Although we did not conduct a gender-specific analysis, it is noteworthy that 68.2% of the participants were women. A study has found that xerostomia is nearly twice as common in women, particularly between the ages of 75 and 85, and that women tend to experience more disabilities as they age, thereby increasing the risk factors for the sensation of dry mouth^24^. Given the mean age of 78 years in our sample, these findings underscore the importance of considering gender when evaluating xerostomia in the elderly.

Speech alterations, including dysarthria and/or apraxia, were associated with a 16% increased risk of bronchoaspiration. Dysarthria is a robust predictor of dysphagia in degenerative and cerebrovascular diseases, affecting 76–90% of patients with these conditions^25^. In our study, 4.7% of participants had Parkinson's disease, consistent with reports that over 80% of these patients develop dysphagia, often leading to aspiration pneumonia^26^. Progressive upper and lower motor neuron involvement in Parkinson's disease and other neuromuscular disorders contributes to oropharyngeal and pharyngeal muscle weakness, resulting in dysarthria and dysphagia^26^. Among post-stroke patients, motor neuron damage impairs essential muscular coordination for speech and swallowing, with dysphagia observed in 32% and dysarthria in 26% of cases^27^. Dysarthria prevalence in our sample was 14%, emphasizing its significant presence in frail elderly individuals. Cognitive function and age also influence speech alteration prevalence (25.6%), affecting temporal aspects, fluency, rhythm, and vocal quality^28^. Buccofacial apraxia, common in dementia patients, correlates with cognitive decline and frailty^29^.

Memory complaints were associated with an 11% increased risk of bronchoaspiration. In our study, 43% of participants reported subjective memory complaints, and 22.1% were diagnosed with cognitive impairment. Previous research supports this link: a study found that 85.9% of elderly individuals with dementia experience oropharyngeal dysphagia, impacting functionality, nutritional status, and increasing respiratory infections and mortality rates. Dementia affects mastication, sensory aspects of swallowing, and is associated with risk factors like malnutrition. The prevalence of oropharyngeal dysphagia (OD) in dementia patients varies by dementia type and severity, influenced by cortical and/or subcortical lesions affecting swallowing neural control^30^. Additionally, frailty, dysphagia, and impaired quality of life are correlated in Alzheimer's patients, irrespective of age, dementia severity, or nutritional status adjustments^31^. These findings emphasize the significance of addressing memory difficulties and cognitive decline in the assessment and management of dysphagia among the elderly.

Participants reporting changes in food consistency had an 8% increased risk of bronchoaspiration. This aligns with studies: one involving 639 LTCF residents found significant links between modified dietary consistency and dysphagia risk, recommending modified textures for safer swallowing^32^. Another study with 883 elderly individuals noted difficulty with thickened liquids and mixed consistencies correlated with oropharyngeal dysphagia risk^33^. In post-stroke patients (≥ 65 years), consistent, thickened food textures (International Dysphagia Diet Standardisation Initiative [IDDSI] classification) were linked to higher malnutrition and prevalence of sarcopenia^34^. These findings highlight the significance of dietary modifications in mitigating dysphagia risks among elderly individuals.

These findings underscore the need for continuous monitoring of these elderly individuals for personalized adjustments in food consistency, aiming to reduce the risk of aspiration pneumonia and malnutrition. Multiple variables, including chronic diseases, falls, urinary incontinence, osteoporosis, hearing impairment, and the use of hearing aids, were found to be associated with complaints of choking among the elderly. These findings align with a meta-analysis indicating that older adults with oropharyngeal dysphagia are significantly more likely to develop urinary incontinence (2.75 times), experience falls (1.47 times), develop sarcopenia (3.10 times), and exhibit frailty (2.66 times) compared to their counterparts without dysphagia^35^. Furthermore, a study involving 522 hospitalized elderly individuals revealed that those with dysphagia and three to four chronic conditions face 2.9 times more difficulties at discharge, a figure that escalates to 8.7 times for those with seven or more conditions^36^.

Although the association between choking and osteoporosis is novel, dysphagia can impede osteoporosis treatment, as patients with dysphagia encounter challenges in medication and supplement ingestion^37^. Additionally, our study identified an intriguing association between choking, hearing loss, and the use of hearing aids, although this correlation lacks substantial supporting evidence. While a study involving 79 healthy elderly individuals suggested that hearing aids do not impact feeding functional capacity^38^, our findings suggest that hearing impairment and the use of hearing aids may exert a more substantial impact on the health and quality of life of the elderly than previously acknowledged.

Our study presented a few limitations. While data collected from medical records offer valuable insights, the presence of incomplete records may pose challenges to result interpretation, potentially affecting the validity and generalizability of our findings. Additionally, relying on self-reported complaints for dysphagia classification, though convenient, introduces subjectivity, influenced by individual health perceptions, possibly leading to inaccurate assessments. Moreover, the study's single-center nature restricts its geographical scope to a specific region of São Paulo, possibly reflecting the characteristics of the participants attending that center. However, despite this limitation, the data offer valuable insights into the elderly population of the Western Zone, aiding in the development of targeted public policies for this demographic group. Notwithstanding these limitations, the study underscores the intricacies of geriatric care and underscores the importance of multidisciplinary approaches in elderly care. It reveals pertinent associations that can inform future clinical practices and health policies. Further research is warranted to broaden these insights, facilitating a more comprehensive understanding and effective management of dysphagia and elderly health needs. This, in turn, can enhance their quality of life and mitigate severe complications such as bronchoaspiration.

## CONCLUSION

These findings underscore the imperative of early detection and a multidisciplinary approach in addressing swallowing disorders among elderly individuals, especially those at heightened frailty levels. By recognizing and addressing these risk factors, healthcare providers can enhance the quality of care and improve the overall well-being of elderly patients. Further research is warranted to expand upon these findings and inform tailored interventions aimed at optimizing swallowing function and reducing associated complications.

## Data Availability

The datasets generated and/or analyzed during the present study are not publicly available due to privacy restrictions, but are available from the corresponding author upon request.
